# Gp120/CD4 Blocking Antibodies Are Frequently Elicited in ART-Naïve Chronically HIV-1 Infected Individuals

**DOI:** 10.1371/journal.pone.0120648

**Published:** 2015-03-24

**Authors:** Jorge Carrillo, Luis Manuel Molinos-Albert, Maria Luisa Rodríguez de la Concepción, Silvia Marfil, Elisabet García, Ronald Derking, Rogier W. Sanders, Bonaventura Clotet, Julià Blanco

**Affiliations:** 1 Institut de Recerca de la SIDA-IrsiCaixa-HIVACAT, Badalona, Barcelona, Spain; 2 Institut de Recerca en Ciències de la Salut Germans Trias i Pujol (IGTP), Hospital Germans Trias i Pujol, Badalona, Barcelona, Spain; 3 Department of Medical Microbiology, Academic Medical Center, University of Amsterdam, Amsterdam, The Netherlands; 4 Department of Microbiology and Immunology, Weill Medical College of Cornell University, New York, New York, United States of America; 5 Universitat Autònoma de Barcelona, Cerdanyola del Vallés, Barcelona, Spain; 6 Universitat de Vic-Central de Catalunya, UVIC-UCC, Vic, Barcelona, Spain; 7 Fundació Lluita contra la SIDA, Badalona, Barcelona, Spain; Pasteur Institute of Shanghai, Chinese Academy of Science, CHINA

## Abstract

Antibodies with the ability to block the interaction of HIV-1 envelope glycoprotein (Env) gp120 with CD4, including those overlapping the CD4 binding site (CD4bs antibodies), can protect from infection by HIV-1, and their elicitation may be an interesting goal for any vaccination strategy. To identify gp120/CD4 blocking antibodies in plasma samples from HIV-1 infected individuals we have developed a competitive flow cytometry-based functional assay. In a cohort of treatment-naïve chronically infected patients, we showed that gp120/CD4 blocking antibodies were frequently elicited (detected in 97% plasma samples) and correlated with binding to trimeric HIV-1 envelope glycoproteins. However, no correlation was observed between functional CD4 binding blockade data and titer of CD4bs antibodies determined by ELISA using resurfaced gp120 proteins. Consistently, plasma samples lacking CD4bs antibodies were able to block the interaction between gp120 and its receptor, indicating that antibodies recognizing other epitopes, such as PGT126 and PG16, can also play the same role. Antibodies blocking CD4 binding increased over time and correlated positively with the capacity of plasma samples to neutralize the laboratory-adapted NL4.3 and BaL virus isolates, suggesting their potential contribution to the neutralizing workforce of plasma *in vivo*. Determining whether this response can be boosted to achieve broadly neutralizing antibodies may provide valuable information for the design of new strategies aimed to improve the anti-HIV-1 humoral response and to develop a successful HIV-1 vaccine.

## Introduction

During the course of an infection process the infected organism develops a broad humoral response, which in conjunction with the innate and T-cell response, control the infection and preserve the integrity of the organism. In the setting of the HIV-1 infection, this humoral response can be detected early during the infection but it is known to be ineffective to control viral replication [1], mainly because the antibodies produced by most patients are nonneutralizing, i. e. recognize viral epitopes that fail to interfere with the replicative cycle of the virus. Although in some patients, antibodies with the capacity to neutralize the autologous virus can be identified after few months of infection, several years are required to develop broadly neutralizing antibodies (bNAbs) [2–4]. To date, many broadly neutralizing antibodies have been identified and all of them recognize epitopes within the HIV-1 envelope glycoprotein (Env) with an important role in viral fitness [2]. These antibodies include CD4 binding site (CD4bs) antibodies (IgGb12 [5] and VRC01 [6]), anti-CD4 induced-epitope antibodies (CD4i antibodies) (X5 [7]), anti-gp41 antibodies (2F5 [8], 4E10 [9] and 10E8 [10]), anti-carbohydrates (2G12 [11]), anti-glycosylated quaternary epitopes (PG9 and PG16) [12] and anti-core antibodies [13]. Among them, antibodies with the ability to block the interaction of gp120/CD4 such as CD4bs antibodies should be highlighted for several reasons: 1) they recognize a conserved region of gp120, 2) they can neutralize a broad number of viral isolates and 3) they can prevent or control the infection in animal models of HIV-1 infection [14,15]. Therefore, the elicitation of this sort of bNAbs is an interesting goal for any vaccination strategy. However, one of the major handicaps in the study of these antibodies is their identification. Broadly neutralizing antibodies in general and CD4bs antibodies in particular, recognize conformational epitopes, which are difficult to mimic *in vitro*. To date, several strategies have been followed to study the CD4bs antibodies, these include the use of recombinant proteins and mutant variants which are differentially recognized by these antibodies [6,16,17]. Also our team has recently developed a cell-to-cell viral transfer assay, which enables the identification of gp120/CD4 blocking antibodies in plasma samples [18]. This assay is based on the viral entry process, which is blocked at early stages in the presence of antibodies that block the gp120/CD4 interaction, like CD4bs antibodies. Furthermore, this work showed a positive correlation between the presence of gp120/CD4 blocking antibodies and the neutralizing capacity of the plasma, being the samples showing this specificity the ones with broader and more powerful neutralizing responses [18]. Interestingly, using a set of recombinant proteins, Lynch et al. have shown that more than 80% of HIV-1 infected patients can develop CD4bs antibodies, indicating that this reactivity might be more frequent than it had been previously described [17]. However, in the latter study a clear correlation between the presence of these antibodies and the neutralizing capacity of the plasma samples was not observed.

To improve the identification of gp120/CD4 blocking antibodies we have developed a competitive flow cytometry-based assay, which enables the detection of these antibodies in plasma or serum samples. This assay is based on the recognition of the Env glycoprotein, expressed on the surface of HIV-1 persistently infected cell lines, by the use of a newly designed huCD4/mouse-IgG (huCD4mIgG) fusion protein. The results showed that the presence of gp120/CD4 blocking antibodies is more represented in HIV-1-chronically infected patients than expected. The levels of these antibodies did not show correlation with the titer of CD4bs antibodies, probably because they may target CD4bs but also other unrelated epitopes. Finally, these antibodies correlate with the neutralizing capacity of plasma samples assayed using laboratory-adapted virus isolates.

## Methods

### Samples

A total of 10 uninfected healthy donors and 36 untreated HIV-1 infected individuals with viral load (VL)>50 copies/mL and with at least two plasma samples separated by one year were selected retrospectively. Main patient’s characteristics are described in Table 1 (mean ± standard deviation). Since it was not possible to establish exactly the date of infection for each patient, time after diagnosis of HIV-1 infection was used as an estimation of infection length. Plasma was prepared by blood centrifugation for 10 minutes at 3000xg.

**Table 1:** Characteristic of ART-naive HIV-1 infected patients.

**n**	36
**%CD4**	28.3±7.1
**CD4/μL**	586±216
**%CD8**	52.2±10
**CD8/μL**	1079±416
**VL (copies/mL)**	51127±183286
**Days after diagnosis**	1803±1933

The data presented here are part of the observational study “ANALYSIS OF HUMORAL RESPONSES IN UNTREATED HIV+ PATIENTS”. The study was carried out at the Hospital Universitari Germans Trias i Pujol (Badalona, Spain) and was approved by the Ethics Committee of the Hospital Universitari Germans Trias i Pujol (PI081306). All participants provided written informed consent to participate in the study.

### Production of the huCD4mIgG recombinant protein

The D1-D2 N-terminal domains of the human CD4 molecule were amplified by standard RT-PCR using the SuperScript III One-Step RT-PCR System with Platinum Taq DNA polimerase (Invitrogen) and the following primers:

CD4 L sense: 5´-CACCATGAACCGGGGAGTCCCTTTTAG-3´ and
CD4L AS NheI: 5´-TATTAGCTAGCACCACGATGTCTATTTTG-3´.

RNA extracted from human peripheral blood mononuclear cells (PBMC) was used as template. The pcDNA3.1huCD4 plasmid was generated after cloning of the CD4 amplimer using the pcDNA3.1 Directional V5-His-TOPO kit and following the manufacturer’s instructions.

The hinge/CH2/CH3 containing-Fc region of murine IgG1 was amplified using the primers:

mIgG1-S: 5´-GAATAGAGCTGGTGGGCTAGCTGTGCCCAGGGATTGTGGT-3´ and
mIgG1-AS: 5´-TTATTCTCGAGTCATTTACCAGGAGAGTGGG-3´.

As template, RNA extracted from the NS1 (ATCC) murine cell line was used.

The amplimer was purified, digested with the FastDigest NheI and XhoI restriction enzymes (Fermentas) and ligated into the pcDNA3.1huCD4 (previously linearized with the same restrictions enzymes) using T4 DNA ligase (Fermentas). The DNA-construct integrity was confirmed by sequencing using the BigDye Terminator v3.1 Cycle Sequencing Kit (Applied Biosystems). To produce the recombinant fusion protein, 293 cells (NIH AIDS reagent program) were transfected with the pcDNA3.1huCD4mIgG1 plasmid using Calphos transfection kit (Clontech) following the manufacturer’s instructions. After 48 hours, the supernatant was collected, clarified by filtration through a 0.45 μm filter (Millipore) and stored at −20°C until use.

### Quantification and titration of supernatants containing the huCD4mIgG recombinant protein

HuCD4mIgG recombinant protein was quantified by an in house ELISA using goat anti-mouse IgG(Fc specific) (Jackson-Immunoresearch) as capture antibody, Rat anti-mouse IgG1 (Biolegend) as secondary antibody and HRP-Goat anti-Rat IgG as detection antibody (Jackson-Immunoresearch). Serial dilutions of purified IgG1-monoclonal antibody clone SK3 (BD Biosciences) was used as standard. Using this approach the concentration of the recombinant protein was equivalent to 11 μg/mL of mouse IgG1. All concentration values of huCD4mIgG fusion protein are given in μg/mL of equivalent mouse IgG1.

HIV-1 NL4.3 chronically-infected MOLT cells [19] were incubated with serial dilutions of the huCD4mIgG-containing supernatant for 30 minutes at room temperature. After washing with PBS, the huCD4mIgG fusion protein bound to gp120 on the infected-cell surface was detected by flow cytometry using a Fc-specific DyLight 649-F(ab)2 Goat anti-mouse IgG (Jackson-Immunoresearch).

### Detection of gp120/CD4 blocking antibodies using a competitive-cytometric assay

HIV-1 NL4.3 and BaL chronically-infected MOLT cells [19] were pre-incubated at room temperature for 25 minutes with serial dilutions of plasma samples or monoclonal antibodies VRC01, VRC03 [6], PG9, PG16 [12], PGT 126 [20] (NIH AIDS Reagent Program), IgGb12, 2G12, 2F5, 447–52D (Polymun), or the polyclonal antibody Goat anti-gp120 (Abcam). Then, the huCD4mIgG-containing supernatant at 0.22 μg/mL was added and the incubation extended for 30 minutes at room temperature. After two washes with PBS, the Fc-specific secondary antibody DyLight 649-F(ab)2 Goat anti-mouse IgG (Jackson-Immunoresearch) was added and incubated again for 15 minutes at room temperature. The cell samples were washed, fixed in 1% formaldehyde (Sigma-Aldrich) and analyzed by flow cytometry.

### Quantification of anti-Env antibodies in plasma samples by flow cytometry

Anti-Env antibodies were quantified as previously described [21]. Briefly, MOLT-NL43, MOLT-BaL and uninfected MOLT cell line were stained with plasma samples at 1/100 dilution, in PBS+10% fetal bovine serum (Life technologies), for 30 minutes at room temperature. After washing with PBS, bound antibodies were detected with a PE-Goat anti-human IgG secondary antibody (Jackson-Immunoresearch). Specific signal was determined as the ratio between the Mean Fluorescent Intensity (MFI) obtained for each infected cell line (NL43 or BaL) and the uninfected one.

### Quantification of anti-Env antibodies in plasma samples by ELISA

Titer of IgG antibodies binding to trimeric Env glycoprotein was determined by ELISA using a D7324-tagged version of the recently described soluble HIV-1 Env trimer BG505 SOSIP.664 gp140 and following the methods described by Sanders et al [22]. Briefly, Maxisorb Elisa plates (Nunc) were coating with D7324 antibody (10 μg/mL) and blocked using TBS+10%FBS for two hours at room temperature. After washing, the BG505 SOSIP.664 protein was added at 1ng/ml in blocking buffer and incubated overnight at 4°C. Then, plates were washed and blocked again using TBS/2% skimmed milk. After washing, plasma samples diluted 1/1000 in TBS/5%FBS/2%skimmed milk were added and incubated for two hours at room temperature. HRP-Goat anti-human IgG (Fc specific) (Jackson-Immunoresearch) was used as secondary antibody. The reaction was revealed using 3, 3′,5, 5′-Tetramethylbenzidine (Sigma-Aldrich) and stopped using 2M of H_2_SO_4_. 2G12 antibody was used as standard and the results are showed as arbitrary units. The IgGb12 antibody, which does not bind to the BG505 SOSIP.664, was used as negative control. Plasma samples were assayed in parallel in D7324 antibody coated and antigen free wells to evaluate background. The signal obtained in these wells was subtracted to the signal obtained with antigen.

### Determination of CD4bs antibodies by ELISA

The presence of CD4bs antibodies was quantified by ELISA using the recombinant protein RSC3 and RSC3Δ371I (RSC3Δ) (NIH AIDS Reagent Program). RSC3 exposes the CD4bs of gp120, and RSC3Δ is a truncated protein with reduced binding of CD4bs antibodies [6,17]. Briefly, 50 ng/well of protein in PBS was bound to 96-well Maxisorb plates (NUNC) overnight at 4°C. After washing and blocking the wells with PBS plus 10% of FBS, plasma samples diluted in blocking buffer were added and incubated overnight at 4°C. 2G12 monoclonal antibody was used as standard and IgGb12, which recognized RSC3 but not RSC3Δ, was used as control of CD4bs antibodies. Anti-CD4bs titer was calculated for each plasma sample as the titer of anti-RSC3 antibodies minus titer of anti-RSC3Δ antibodies (RSC3- RSC3Δ).

### Neutralization capacity of plasma samples

Neutralization assays were as described [18]. Plasma samples, inactivated for 1 hour at 56°C, were used to evaluate virus-specific neutralization activity, using the TZM-bl neutralization assay [23]. Briefly, three-fold serial dilutions of plasmas were incubated in 96-well plates in duplicates for 1 hour with 200 TCID50 of two laboratory-adapted viral strains (HIV-1 NL4–3 and BaL) and two primary isolates (AC10 and SVPB16). 10^5^ TZM-bl cells were added per well and incubated for 48 hours at 37°C and 5% CO_2_. Luciferase activity was determined using the BriteLite plus (PerkinElmer) and the luminescence was then quantified in a luminometer (Fluoroskan Ascent FL, Labsystem). The relative light units (RLU) obtained were used to calculate the percentage of neutralization using the formula: % Neutralization = [1−(R−Rcc/Rvc)] × 100; where R = RLU of the tested plasma, Rcc = RLU of cell alone and Rvc = RLU of virus alone. The dose inducing 50% of total inhibitory capacity (IC50) was calculated and results are shown as reciprocal dilution.

### Statistic analysis

Data were analyzed using *GraphPad Prism* and R (v.3.1.2) softwares. Fisher´s exact test, Mann-Whitney U test, Paired t-test and Spearman´s correlation were applied when required.

## Results

### High prevalence of gp120/CD4 blocking antibodies in ART-naïve HIV-1 chronically infected patients

Antibodies showing the capacity to block the interaction between gp120 and the CD4 receptor expressed on the surface of CD4+ T cells may be considered as putative neutralizing antibodies. To determine the presence of this kind of antibodies in our cohort of HIV-1 infected patients, we developed a flow cytometry-based competitive assay using a human-CD4/mouse-IgG fusion protein and two HIV-1-chronically infected MOLT cell lines (BaL and NL4.3). HIV-1 Env can be detected on the surface of these cells by anti-gp120 antibodies that recognize conformational-restricted trimer-specific (PG9, PG16) and non-conformational restricted epitopes, such as IgG2G12, PGT126 or CD4bs antibodies (IgGb12, VRC03 and VRC01), with similar results (S1 Fig.). Moreover, antibodies targeting epitopes that are poorly exposed (such as MPER) or only exposed after gp120/CD4 interaction (CD4-induced) showed low or negligible signal, respectively (S1 Fig.). Despite differences among the former staining due to different affinity/avidity of antibodies, the results strongly suggested that the chronically-infected MOLT cell lines mainly expressed a functional-native Env glycoprotein on their surface (S1 Fig.). Similar results were obtained with MOLT-BaL (data not shown).

**Fig 1:**
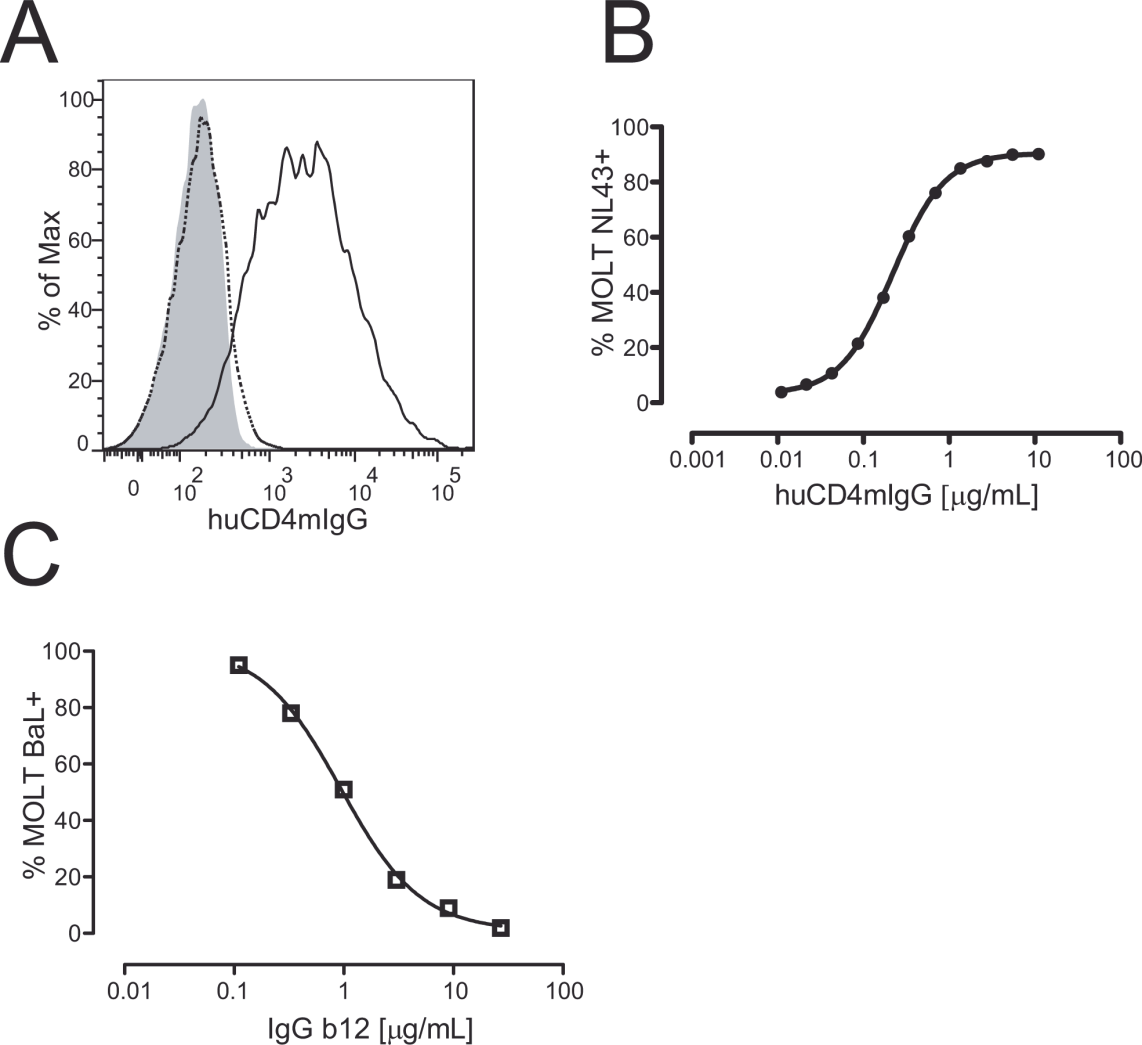
Identification of gp120/CD4 blocking antibodies. The presence of antibodies with the capacity to block the interaction between CD4 and gp120 was evaluated with a flow cytometry competitive assay using a chronically infected cell line and a huCD4mIgG recombinant protein. A) MOLT-NL4.3 (black line) and uninfected-MOLT cells (gray) were stained with huCD4mIgG (10μg/mL) and a DyLight-649 Fab2 Goat anti-mouse IgG as secondary antibody. Dotted line shows the staining of MOLT-NL43 cell using a mouse IgG1 isotype control. B) MOLT-NL4.3 cells were stained with serial dilutions of huCD4mIgG. C) IgGb12 inhibition of the binding of huCD4mIgG to MOLT-NL4.3.

**Fig 2:**
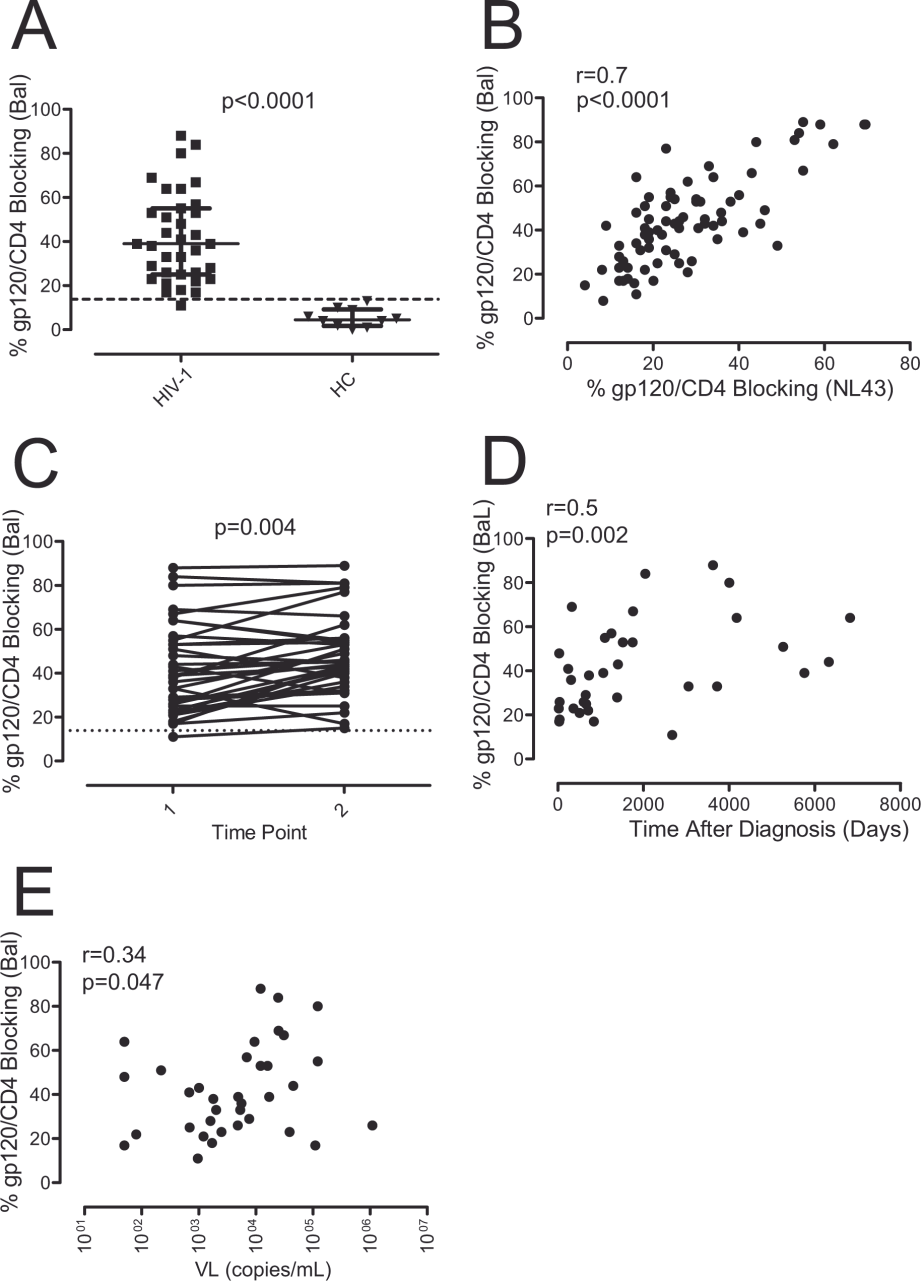
Gp120/CD4 blocking antibodies in ART-naive HIV-1 infected patients. A) The presence of gp120/CD4 blocking antibodies was determined in the plasma of 35 ART-naive HIV-1 infected patients (squares) and compared with 10 uninfected healthy control individuals (HC, triangles). The cytometric competitive assay was performed using the huCD4mIgG recombinant protein and the MOLT-BaL cell line. Differences were calculated using Mann-Whitney U test. Dotted line shows cut-off of positivity calculated as mean+2xSD of HC samples. B) Correlation between levels of gp120/CD4 blocking antibodies, using MOLT-BaL and MOLT-NL4.3 cells, was determined by Spearman´s test. C) Gp120/CD4 blocking antibodies were determined in two plasma samples separated by one year from the same HIV-1 infected individuals (paired t-test). Dotted line shows cut-off of positivity as in panel A. D and E) Correlation analysis between the percentage of gp120/CD4 blocking activity of plasma samples and time after diagnosis or viral load (VL) was performed using Spearman´s correlation.

MOLT infected cell lines could also be stained with the huCD4mIgG recombinant protein (Fig. 1A and B) and the addition of the IgGb12 antibody, which recognizes the CD4 binding site in gp120, blocked the interaction between the huCD4mIgG and gp120 in a concentration dependent way, indicating that this inhibition could be used as a measure of the presence of antibodies directed against the CD4 binding site of gp120 (Fig. 1C). Following this approach, we determined the prevalence of gp120/CD4 blocking antibodies in plasma samples from ART-naïve HIV-1 infected individuals. When BaL chronically-infected MOLT cell lines were used, gp120/CD4 blocking antibodies were detected in 34 out of 35 of samples tested (97%, Fig. 2A), and showed a good correlation with the gp120/CD4 blocking antibodies detected using MOLT-NL43 (r = 0.7, p<0.0001) (Fig. 2B).

When plasma samples obtained from the same patients after one year of untreated infection were analyzed, statistically significant differences were found between both time points, supporting that the presence of these antibodies increased over time (p = 0.004) (Fig. 2C). According to that, the presence of this kind of antibodies correlated with the time after diagnosis (Fig. 2D) indicating that a prolonged exposure to the virus may drive the development of these antibodies. However, a poor correlation was found between the presence of gp120/CD4 blocking antibodies and the viral load (r = 0.34, p = 0.047) (Fig. 2E), suggesting that viremia may not be a major factor for the development and or maintenance of these antibodies although a certain level of virus may be required.

### Detection of CD4bs antibodies in plasma samples by ELISA

**Fig 3:**
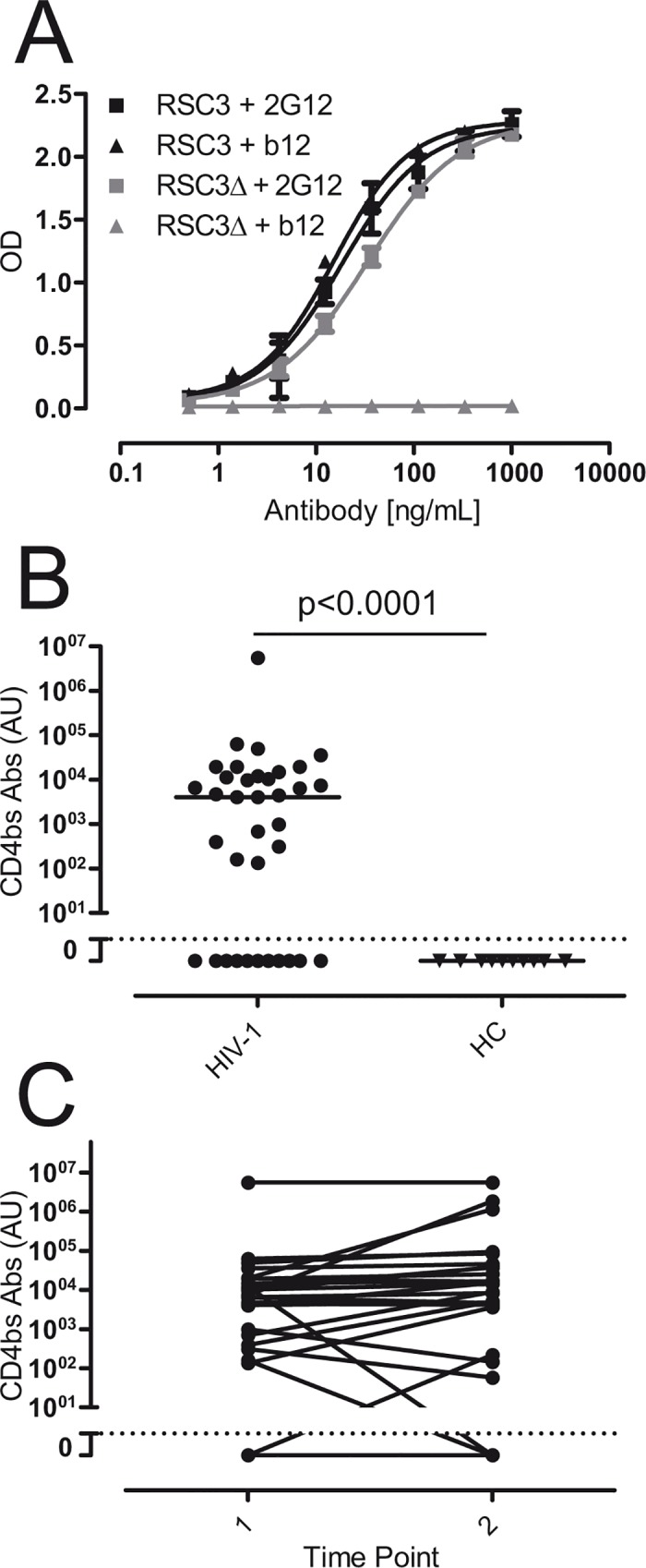
Detection of CD4bs antibodies by ELISA. The presence of CD4bs antibodies in ART-naive HIV-1 infected patients (HIV-1) was evaluated by ELISA using the RSC3 and the RSC3Δ371I (RSC3Δ) recombinant proteins. A) Standard curve was calculated using the 2G12 antibody, which recognized both recombinant proteins. The antibody IgGb12, which recognized RSC3 but not RSC3Δ, was used as control. B) The titer of CD4bs antibodies was calculated as RSC3-RSC3Δ. Differences with uninfected healthy donor controls (HC) were evaluated by Fisher´s exact test. Dotted line show cut-off of positivity calculated as mean+2xSD of HC samples. C) Levels of CD4bs antibodies were determined in two plasma samples separated by one year from the same HIV-1 infected individuals. Dotted line indicated the cut-off as in panel B. No difference between both time points was observed (paired t-test).

Given that CD4bs antibodies are able to block the interaction between gp120 and CD4, the presence of these antibodies was quantified by ELISA. Each plasma sample was assayed against a protein that exposes the CD4bs and can be recognized by CD4bs antibodies (RSC3); and a mutated version of this protein (RSC3Δ) which shows an impaired reactivity for most of CD4bs antibodies (Fig. 3A) [6,17]. Therefore, results obtained after subtracting anti-RSC3Δ titers to anti-RSC3 titers were used as an estimation of CD4bs antibodies. The results showed that 25 out of 36 analyzed patients (70%) showed this sort of antibodies whereas none of the healthy uninfected controls were positive (p<0.0001, Fisher´s exact test) (Fig. 3B). No correlation was observed between CD4bs antibodies titer, VL, CD4+ T cells count and time from diagnosis (data not shown). To determine the stability of the presence of these antibodies, we quantified the CD4bs antibodies in subsequent samples from the same patients in the absence of antiretroviral therapy (Fig. 3C). No differences were observed between both time points, suggesting that this kind of antibodies is generally maintained in chronic infection, at least for one year. Just one patient developed and other two become undetectable for the presence of CD4bs antibodies after one year, indicating some extent of variability among individuals. These results confirm that CD4bs antibodies are commonly generated in HIV-1 chronically infected patients and are in line with previously published data[17].

### CD4bs antibodies are not correlated with gp120/CD4 blocking activity of plasma

**Fig 4:**
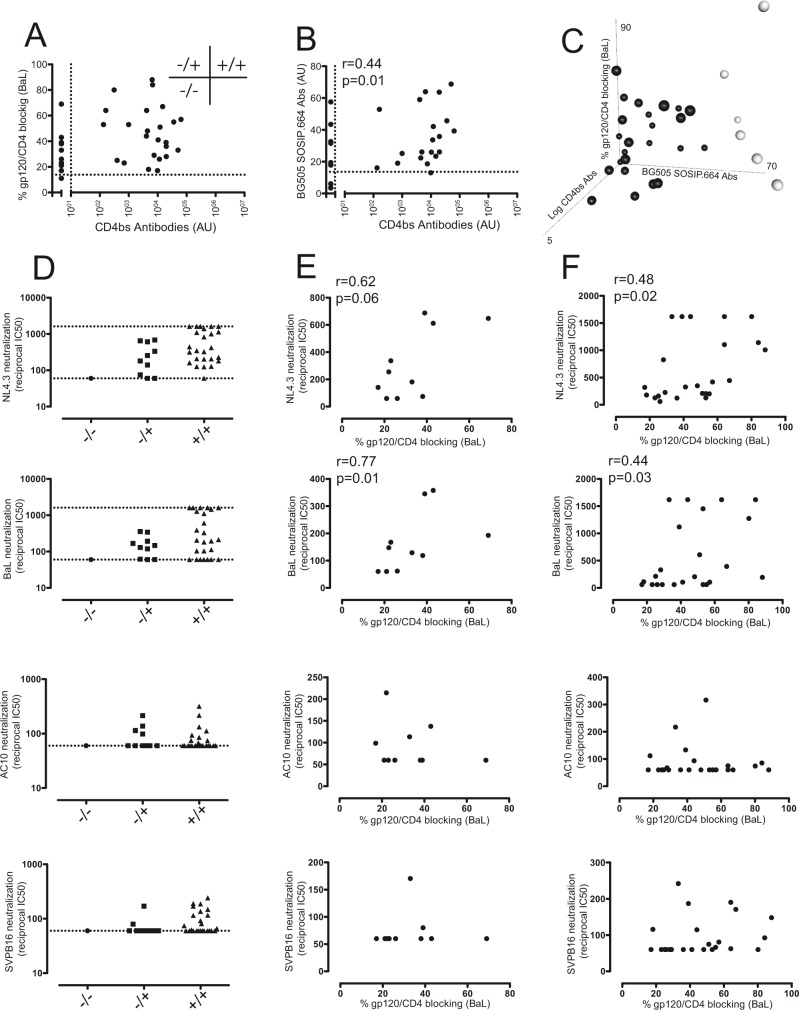
Correlation of gp120/CD4 blocking antibodies and CD4bs antibodies with the neutralizing capacity of plasma samples. A) Gp120/CD4 blocking activity showed no correlation with the titer of CD4bs antibodies (Spearman´s correlation). Dotted line indicated the positivity cut-off of each parameter. Three groups of plasma samples were defined using cut-off values: plasma samples showing CD4bs and gp120/CD4 blocking antibodies (+/+), samples with detectable gp120/CD4 blocking Abs but undetectable CD4bs Abs (-/+) and plasma samples lacking both specificities (-/-).B) Titer of BG505 SOSIP.664 reactive antibodies showed a weak positive correlation with CD4bs Abs. Dotted line indicated the positivity cut-off of each parameter. Note that most of plasma samples negative for CD4bs Abs showed Env-reactive antibodies. C) gp120/CD4 blocking activity was plotted against the titer of BG505 SOSIP.664 binding antibodies and titer of CD4bs antibodies. Six plasma samples showing high reactivity against the trimer and variable levels of CD4bs antibodies and gp120/CD4 blocking activity are outlined (clear symbols). D) Neutralizing capacity of plasma samples were assayed using four viral isolates: NL4.3, BaL, AC10 and SVPB16. Samples were classified according to panel A. Dotted lines indicate upper and lower dilutions of plasma samples assayed. E) Samples lacking CD4bs Abs but showing gp120/CD4 blocking Abs (-/+) and F) samples showing both specificities (+/+) were analysed for correlation between gp120/CD4 blocking activity and neutralizing capacity (reciprocal IC50) using the viral isolates described in D. Spearman´s correlation analysis was performed (r and p values are only shown for significant correlations).

To determine whether CD4bs antibodies were linked to gp120/CD4 blocking activity, a correlation analysis was performed. The results did not show any correlation between both parameters (r = 0.2612, p = 0.1296, Fig. 4A). Some plasma samples with high titers of CD4bs antibodies showed a poor gp120/CD4 blocking activity suggesting that these antibodies may be nonneutralizing antibodies. On the other hand, a subset of samples (28%, 10 out of 36) did not apparently contain CD4bs antibodies but were able to prevent the binding of gp120 to its receptor, suggesting that CD4bs antibodies that are able to recognize equally both RSC3-based proteins or antibodies elicited against other region than CD4bs can also block the interaction of gp120 with its receptor. To evaluate these options, we quantified by ELISA the presence of antibodies that recognize the trimeric Env protein using the soluble trimer BG505 SOSIP.664 gp140. 27 out of 31 HIV-1+ plasma samples analyzed were positive. The mean+2xSD of the signal obtained with plasma samples from uninfected healthy control was used as positivity cut-off (S2 Fig.). The titer of antibodies against the BG505 SOSIP.664 trimer showed a slight positive correlation with CD4bs antibodies (Spearman´s r = 0.421, p = 0.02) (Fig. 4B). Similar results were obtained when the anti-Env humoral response was quantified by flow cytometry using MOLT-BaL cells (S2 Fig.), reinforcing that the Env glycoprotein expressed on the surface of these cells is mainly trimeric. Interestingly, most of plasma samples lacking CD4bs Abs were trimer-positive and showed some gp120/CD4 blocking activity; indicating that specificities other than the CD4bs may mediate this action. In addition, when all three parameters (trimer-specific Abs, CD4bs Abs titer and gp120/CD4 blocking activity) were plotted altogether six plasma samples stood out that showed high BG505 SOSIP.664 trimer recognition (Fig. 4C). Of them, only two showed CD4bs antibodies and high potency to block the gp120 binding to CD4, suggesting that this plasma samples may contain high titers of neutralizing CD4bs antibodies. In contrast, 3 out of these 6 samples showing high recognition of the trimer and CD4bs Ab titer failed to efficiently block the binding of CD4 to trimeric Env, suggesting that CD4bs antibodies present in these samples may be poorly neutralizing. A last sample lacked CD4bs antibodies but showed high anti-trimer titer and high gp120/CD4 blocking activity, suggesting that antibodies other than CD4bs are responsible of gp120/CD4 blocking activity of this sample.

### Plasma samples with gp120/CD4 blocking antibodies showed neutralizing capacity

To test whether plasma samples containing gp120/CD4 blocking antibodies showed an improved neutralizing capacity, plasma samples were classified according to the presence of this sort of antibodies and CD4bs antibodies, and their neutralizing capacity assayed by a TZM-bl based neutralization assays. Similar results were obtained when plasma samples were classified according to BG505 SOSIP.664 binding and CD4bs Abs titer (Fig. 4B and data not shown). As a reference, four different HIV-1 viral isolates were used: two laboratory adapted (BaL and NL4.3) and two primary isolates (AC10 and SVPB16). As expected, most of the samples showed neutralization capacity (reciprocal IC50>60) against BaL (72%, 26 out of 36) and NL4.3 (89%, 32 out of 36), whereas only some of them could neutralize SVPB16 (39%, 14 out of 36) and AC10 (36%, 13 out of 36) but with poor potency as denote the low reciprocal IC50 of plasma dilution (Fig. 4D). To investigate the relative contribution of gp120/CD4 blocking antibodies to neutralizing capacity of plasma, a correlation analysis between both parameters was performed for each group (Fig. 4E and F). The results showed a positive correlation between the presence of gp120/CD4 blocking antibodies and the neutralizing capacity of plasma samples for NL4.3 and BaL isolates, indicating that these antibodies may contribute to the neutralizing workforce of plasma, even though with low amplitude. Interestingly, some plasma samples showing a high gp120/CD4 blocking capacity showed a poor neutralizing activity, suggesting that other factors, such as sequence variation of target epitopes, might modulate the inhibition activity of these antibodies.

### Gp120/CD4 blocking activity is mediated by anti-gp120 defined specificities

**Fig 5:**
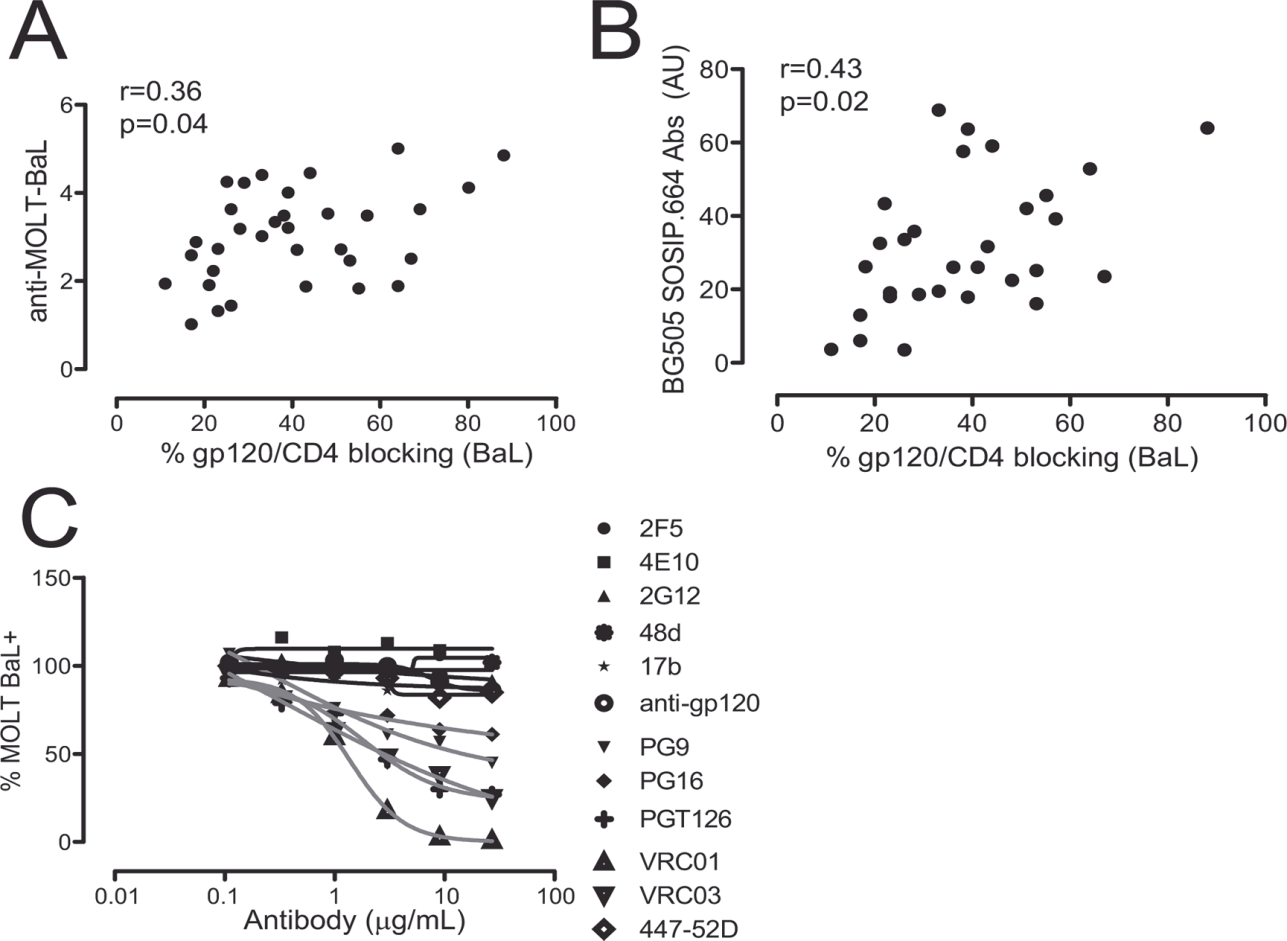
Specificity of gp120/CD4 blocking antibodies. A) The IgG response against Env was evaluated by flow cytometry using MOLT-BaL and uninfected-MOLT cell line. The levels of anti-Env IgG antibodies were calculated as MFI ratio (MOLT-BaL/MOLT). B) Titer of antibodies against the soluble trimer BG505 SOSIP.664 gp140 was determined by ELISA. A correlation analysis between the anti-Env levels and gp120/CD4 blocking activity is shown (Spearman´s correlation). C) A set of anti-Env antibodies, whose reactivity is well-known, was used to evaluate the specificity of the antibodies that were able to block the binding of the huCD4mIgG recombinant protein to MOLT-BaL cells. The antibodies tested included CD4bs Abs (VRC01, VRC03), anti-glycosylated epitopes (2G12, PGT126), anti-glycosylated-quaternary epitopes (PG9, PG16), CD4 induced epitopes (17b,48d), anti-gp41 (2F5, 4E10) and a polyclonal goat anti-gp120 (anti-gp120) obtained by immunization using rgp120.

In order to determine whether the gp120/CD4 blocking activity of plasma samples may be mediated by a polyclonal Env-specific humoral response, a correlation analysis between the gp120/CD4 blocking activity and anti-Env IgG antibodies was performed. Anti-Env IgG was determined by flow cytometry using MOLT-BaL or MOLT-NL4.3 (not shown) and MOLT uninfected cells, and the specific signal expressed as MFI ratio. In addition, anti-Env IgG were also evaluated by ELISA using the soluble trimer BG505 SOSIP.664. The results showed a positive but modest correlation between the gp120/CD4 blocking capacity of plasma and anti-Env response suggesting again that the blocking activity would be mediated by defined antibodies and not simply by an Env-specific polyclonal response (Fig. 5A and 5B). To better characterize the specificity of the gp120/CD4 inhibition, several well-characterized antibodies were assayed in the same conditions (Fig. 5C). In addition to IgGb12, other antibodies directed against the CD4 binding site in gp120, such as VRC01, showed blocking activity. The PGT126 antibody, which recognize an epitope dependent on V3 loop and glycosylation at position 332/301 within gp120 [20], and PG9/PG16, which recognize a glycosylated-quaternary epitope strongly dependent on the native trimeric structure of the Env protein [12], also showed blocking capacity that was lower than IgGb12 or VRC01 antibodies. However, neutralizing antibodies such as 2G12, 447–52D, 2F5 and 4E10; and non-neutralizing polyclonal antibodies directed against other epitopes in gp120, failed to block the interaction between gp120 expressed on the surface of the cells and the huCD4mIgG recombinant protein. These results suggested that the gp120/CD4 blocking antibodies detected in plasma from HIV-1 infected patients may be the result of some defined specificities, mainly CD4bs antibodies but also other antibodies such as PGT126, PG16 or PG9-like, and cannot be explained by a polyclonal humoral response against HIV-1.

## Discussion

Despite it is widely accepted that the humoral immune response is not able to clear HIV-1 infection once started, some patients develop broadly neutralizing antibodies whose efficacy protecting against the infection has been widely reported in defined settings. It has been shown that this kind of antibodies can protect against new infection in non-human primate models [24–28] and help to control viremia in chronically infected patients [29]. These observations make the development of this kind of antibodies one of the major goals of any vaccination strategy [2].

Although our knowledge on neutralizing antibodies has improved during the last years with the identification of new specificities, many questions still remain unresolved such as why they are only elicited in a restricted set of patients or why they take so much time to appear. One intrinsic characteristic of bNAb is that they recognize epitopes that play a major role in the viral fitness. Quite often they are conformational and in many cases depending on glycosylation and masked by the three dimensional structure of the envelope glycoprotein, only being accessible to the immune system after conformational changes of the envelope glycoprotein. Among the susceptible epitopes to be targeted by bNAbs, the CD4 binding site of gp120 is a valuable region since it plays a major role in HIV-1 infection, it is highly conserved and remains exposed to its ligand. Actually, some of the most powerful broadly neutralizing antibodies recognize this domain in gp120 (IgGb12, VRC01, PGV04 and others). The CD4bs of gp120 is a conformational domain, which comprises a set of amino acids from the C2, C3, C4, V5 and C5 regions of gp120 [30]. HIV-1 infected patients develop CD4bs antibodies quite often; however, not all of them are broadly neutralizing indicating that the fine specificity of the antibody and/or the affinity for the ligand may be important to confer neutralizing properties to the antibody molecule [17]. In this way, the main difference between neutralizing and nonneutralizing CD4bs antibodies seems to be the capacity of the former to recognize the CD4bs in its trimeric-native conformation with high affinity [22,31]. Nonneutralizing CD4bs antibodies are able to block the interaction between CD4 and monomeric gp120 but are ineffective at blocking the binding to the native envelope glycoprotein since they are not able to bind to the trimeric protein [22], probably due to the conformational masking of the receptor binding domain of gp120 [32].

To evaluate the frequency of neutralizing CD4bs antibodies in HIV-1 infected individuals we have compared binding to core proteins exposing the CD4bs, binding to soluble and membrane expressed trimeric Env glycoproteins and functional ability to block CD4 binding in plasma samples from HIV-1 infected patients. We have developed a competitive flow cytometry-based functional assay that enables the identification of gp120/CD4 blocking antibodies in the context of a native envelope glycoprotein expressed on the surface of HIV-1-infected cells. This assay is based on the capacity of plasma samples to block the binding of a recombinant huCD4mIgG fusion protein to Env proteins expressed on the surface of infected cells. Using this assay we have been able to determine that the presence of gp120/CD4 blocking antibodies is very common among untreated HIV-1 chronically-infected patients. About 97% of patients showed gp120/CD4 blocking antibodies and this functional activity correlated with the recognition of the soluble and membrane expressed native glycoprotein, even though both came from different HIV-1 strain (clade A and B). Although neutralizing CD4bs antibodies are able to carry out this blocking activity, the titer of CD4bs antibodies and the gp120/CD4 blocking activity did not show significant correlation. This lack of association indicated that not all CD4bs antibodies can efficiently inhibit the interaction between gp120 and CD4. Actually, some patients with high titer of CD4bs antibodies showed poor gp120/CD4 inhibition activity indicating that these antibodies might be nonneutralizing antibodies. Conversely, some plasma samples showing undetectable levels of CD4bs antibodies were able to block the interaction between gp120 and its receptor, indicating that CD4bs antibodies that are able to recognize both RSC3 and RSC3Δ proteins or antibodies elicited against other epitopes may also show blocking capacity. Actually, most of these plasma samples showed IgGs that were able to bind to trimeric Env glycoprotein. In this line, the PGT126, PG9 and PG16 bNAbs, despite not recognizing the CD4 binding site, showed a certain degree of gp120/CD4 blocking activity, probably in an allosteric way. A similar mechanism has been also postulated for the PGT121 antibody [33]. Whereas antibodies such as 2G12 or anti-V3, which recognize other epitopes than the CD4bs in gp120, blocked the virus binding to target cells [34,35]; they failed to block CD4mIgG binding to trimeric Env. In addition, other antibodies, such as 2F5 and 4E10 (anti-MPER) or 48d and 17b (CD4 induced epitopes), were also unable to block the interaction between huCD4mIgG and gp120. A similar lack of inhibition was observed using a goat anti-gp120 polyclonal antibody generated by immunization with recombinant gp120. These data and the correlation observed with total anti-Env antibodies strongly suggest that the gp120/CD4 blocking activity detected in plasma samples from ART-naïve HIV-1 infected patients are the result of definite specificities rather than a polyclonal humoral response against Env. Interestingly, gp120/CD4 inhibition in our assay correlated with the neutralizing capacity of plasma samples measured as reciprocal IC50 of neutralization for both NL4.3 and BaL isolates but not for the primary isolates AC10 and SVBP16. Altogether, the results indicated that these antibodies may contribute to neutralizing capacity of plasma but an important fraction of them are not cross-reactive.

Although the titer of these antibodies, measured as the percentage of gp120/CD4 inhibition, showed a poor correlation with viral load, patients with a viral load over 5000 copies/mL showed the highest titers (data not shown), suggesting that certain amount of antigen should be needed for generating this sort of antibodies and/or that the virus of these patients are resistant to the action of the autologous neutralizing antibodies as it has been previously shown [36,37]. In addition, a positive correlation with the time after diagnosis was observed and when a one-year after samples were analyzed, they showed an increase titer of these antibodies supporting the idea that gp120/CD4 blocking antibodies, like neutralizing antibodies, increase over the course of infection.

It has been shown that HIV-1 infection induces a drastic alteration in the biology of B cells, affecting mainly to the memory subset. The loss of these cells correlates with the lack of protective antibodies against vaccines and recall pathogens in HIV-1 infected patients [38]. Interestingly, in our study the titer of these antibodies seem to be stable at least during one year, strongly suggesting that memory humoral response is preserved in these patients, at least with regard to this specificity. To determine the specificity of these antibodies, which cells are responsible for their production and whether viremia could contribute to maintain them are important questions that need further studies.

## Conclusions

The present study shows that almost all HIV-1 infected patients can develop antibodies that are able to block the binding of gp120 to its receptor. These antibodies include not only those elicited against the CD4bs but also those recognizing a limited number of other unrelated envelope epitopes. These latter antibodies may play a key role in the neutralizing capacity of plasma although, in some cases, with modest amplitude. To improve our knowledge about this sort of antibodies would be greatly helpful for the design of new immunogens which would be able to drive the maturation of these antibodies towards the acquisition of broadly neutralizing abilities.

## Supporting Information

S1 Fig MOLT T cells chronically infected with HIV-1 express trimeric native Env glycoproteins. MOLT-NL4.3 cells were stained with antibodies targeting several epitopes within Env glycoprotein. Despite some antibodies such as CD4bs Abs, 2G12 or PGT126 (A and B) are able to recognize trimeric and monomeric but properly glycosylated protein, respectively; the PG9 and PG16 antibodies recognize only native trimeric Env protein (C).The staining obtained using all of them are comparable, pointing out that the structure of the Env protein on the surface of chronically infected MOLT cells is mainly native. This statement is supported by the negligible signal obtained using antibodies that recognize epitopes exposed only after gp120/CD4 interaction such as 17b and 48d (D), and the low signal achieved using antibodies elicited against poor expose epitopes such as anti-MPER antibodies (2F5)(E).
(TIF)

S2 Fig Plasma samples from HIV-1 infected individuals contain antibodies that bind to trimeric Env glycoproteins. A) IgG response against Env-trimeric glycoprotein was evaluated by ELISA, using the newly generated soluble trimer BG505 SOSIP.664 gp140, and B) by flow cytometry, using the MOLT-BaL and uninfected MOLT cells. Specific signal was calculated as the ratio between the MFI obtained with both cell lines. Dotted line shows the positive cut-off that was calculated as mean+2xSD of the values obtained with plasma samples from uninfected healthy controls. Mann-Whitney test was applied. C) Spearman´s correlation analysis between the antibody titers calculated in A and B showed a weak but positive correlation between both parameters. Note the BG505 SOSIP.664 is subtype A and BaL is subtype B.
(TIF)
