# Supplementary figures and images for: Gp120/CD4 Blocking Antibodies Are Frequently Elicited in ART-Naïve Chronically HIV-1 Infected Individuals
Source: PLoS One. 2015 Mar 24;10(3):e0120648. doi: 10.1371/journal.pone.0120648 (PMC4372395; doi:10.1371/journal.pone.0120648)

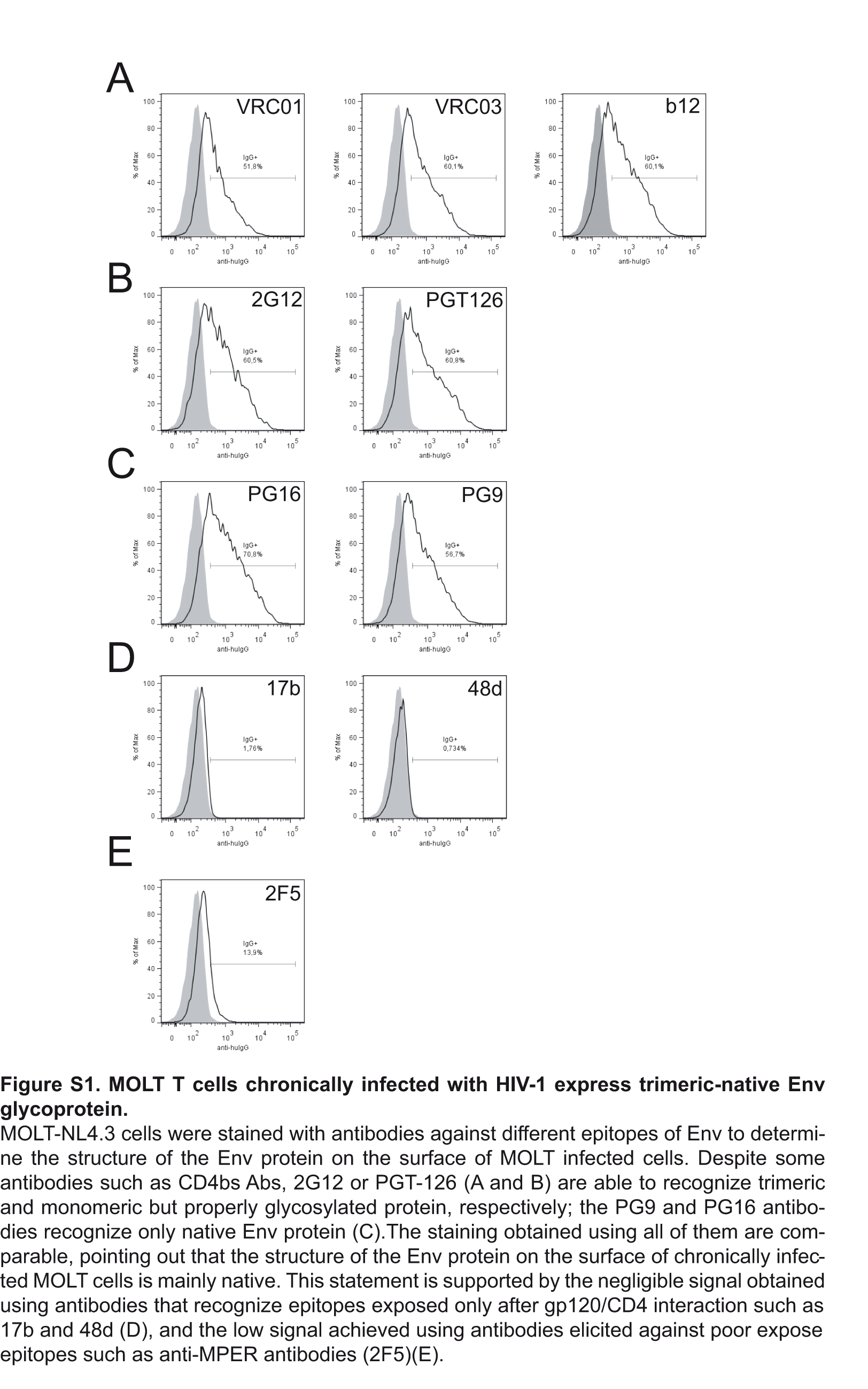

Supplement: S1 Fig — MOLT-NL4.3 cells were stained with antibodies targeting several epitopes within Env glycoprotein. Despite some antibodies such as CD4bs Abs, 2G12 or PGT126 (A and B) are able to recognize trimeric and monomeric but properly glycosylated protein, respectively; the PG9 and PG16 antibodies recognize only native trimeric Env protein (C).The staining obtained using all of them are comparable, pointing out that the structure of the Env protein on the surface of chronically infected MOLT cells is mainly native. This statement is supported by the negligible signal obtained using antibodies that recognize epitopes exposed only after gp120/CD4 interaction such as 17b and 48d (D), and the low signal achieved using antibodies elicited against poor expose epitopes such as anti-MPER antibodies (2F5)(E). (TIF) [file pone.0120648.s001.tif]

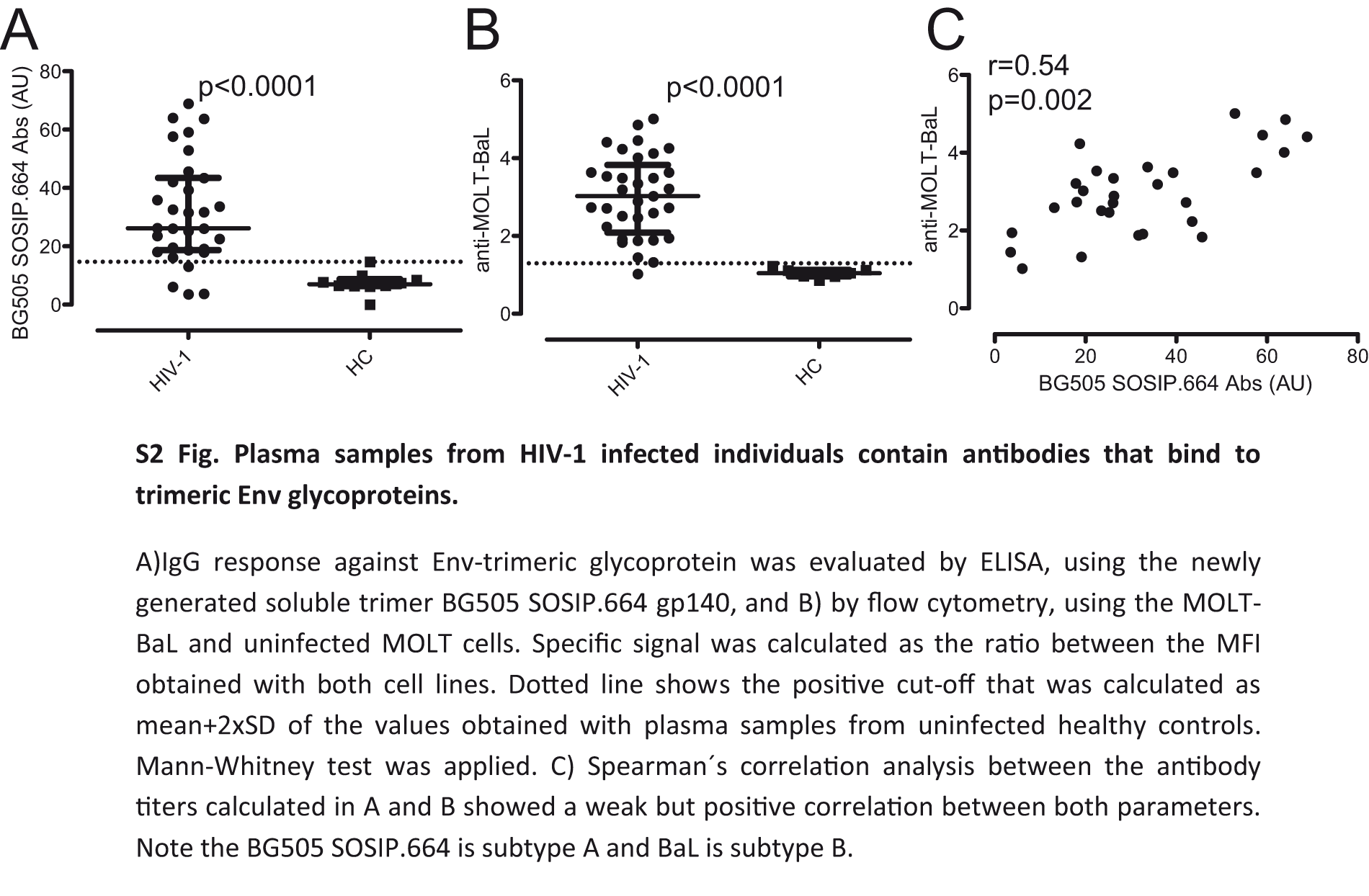

Supplement: S2 Fig — A) IgG response against Env-trimeric glycoprotein was evaluated by ELISA, using the newly generated soluble trimer BG505 SOSIP.664 gp140, and B) by flow cytometry, using the MOLT-BaL and uninfected MOLT cells. Specific signal was calculated as the ratio between the MFI obtained with both cell lines. Dotted line shows the positive cut-off that was calculated as mean+2xSD of the values obtained with plasma samples from uninfected healthy controls. Mann-Whitney test was applied. C) Spearman´s correlation analysis between the antibody titers calculated in A and B showed a weak but positive correlation between both parameters. Note the BG505 SOSIP.664 is subtype A and BaL is subtype B. (TIF) [file pone.0120648.s002.tif]
